# Targeting the circadian modulation: novel therapeutic approaches in the management of ASD

**DOI:** 10.3389/fpsyt.2024.1451242

**Published:** 2024-10-11

**Authors:** Yuxing Zhang, Yinan Chen, Wu Li, Liya Tang, Jiangshan Li, Xiang Feng

**Affiliations:** ^1^ School of Acupuncture, Tuina and Rehabilitation, Hunan University of Chinese Medicine, Changsha, Hunan, China; ^2^ McGovern Medical School, The University of Texas Health Science Center at Houston, Houston, TX, United States

**Keywords:** circadian clock, neurodevelopmental disorders, autism spectrum disorder, therapeutic approaches, chronotherapies

## Abstract

Circadian dysfunction is prevalent in neurodevelopmental disorders, particularly in autism spectrum disorder (ASD). A plethora of empirical studies demonstrate a strong correlation between ASD and circadian disruption, suggesting that modulation of circadian rhythms and the clocks could yield satisfactory advancements. Research indicates that circadian dysfunction associated with abnormal neurodevelopmental phenotypes in ASD individuals, potentially contribute to synapse plasticity disruption. Therefore, targeting circadian rhythms may emerge as a key therapeutic approach. In this study, we did a brief review of the mammalian circadian clock, and the correlation between the circadian mechanism and the pathology of ASD at multiple levels. In addition, we highlight that circadian is the target or modulator to participate in the therapeutic approaches in the management of ASD, such as phototherapy, melatonin, modulating circadian components, natural compounds, and chronotherapies. A deep understanding of the circadian clock’s regulatory role in the neurodevelopmental phenotypes in ASD may inspire novel strategies for improving ASD treatment.

## Introduction

Autism spectrum disorder (ASD) is a cluster of early neurodevelopmental conditions characterized by deficits in social communication, repetitive and stereotyped behaviors, as well as atypical sensory processing (1, 2). The latest Global Burden of Disease Study (GBD) reports that ASD diagnoses have increased significantly in recent decades in many countries, including China, with a global prevalence estimated at 1.2% to 2% (3, 4). This increase is largely attributable to changes in the diagnostic criteria for ASD, including their broadening, as well as enhanced public awareness and improved recognition of the disorder by healthcare professionals. Individuals with ASD may experience barriers to social interaction, economic activity, and well-being (5). The impacts exhibit varying magnitudes, encompassing but not limited to effects on educational and professional accomplishments, mental well-being, familial and societal roles (6, 7). ASD often imposes a substantial familial and socioeconomic burden, necessitating the provision of assistance to adults who lack independent functioning. This leads to escalated healthcare and educational expenses, as well as financial losses for caregivers (8).

Over the past eight decades, neuroscientists and clinicians have been captivated by various aspects of ASD, including risk factors, diagnostic criteria, therapeutic options, mechanistic studies, genomic patterning investigations, and societal influences. Currently, there are various treatment strategies for ASD, which can be broadly divided into two types, nonpharmacological treatment and pharmacological treatment (9, 10). The primary focus of nonpharmacological approaches lies in the early implementation of intensive behavioral intervention, with Applied Behavior Analysis (ABA) serving as the central area of interest and encompassing various iterations (11). In addition to ABA, current treatment strategies also include therapies targeting specific symptoms, such as occupational therapy and speech and language therapy. Moreover, a variety of pharmacological interventions, including guanfacine, SSRIs, stimulants, and anti-anxiety treatments, are employed to manage specific symptoms associated with ASD. Several randomized controlled trials indicated that low-intensity interventions from parents, including establishment of joint engagement, shared attention, and balanced play, which encourage children to take more initiative contribute to better social behavior and communication (12). While the results of other studies on low-intensity interventions are not satisfactory, this may be related to individual differences (13, 14), strategies that for most children may not be effective for every child. Unfortunately, the lack of molecular target hinders the development of new drugs for autism. Therefore, formulating strategies to regulate or even block the processes involving key signal molecule transduction, epigenetics, and molecular modification in the pathogenesis of autism is a crucial research direction.

Circadian rhythms, universally recognized as essential for maintaining overall health, are driven by the internal master clock through circadian transcriptional-translational feedback loops (TTFLs).These loops are composed of *CLOCK*(Circadian locomotor output cycles kaput or *NPAS2*)–*BMAL1* (brain and muscle ARNT-like protein 1, also called *MOP3*) transcriptional activators and *CRY*(cryptochrome)–*PER*(period) transcriptional repressors (15, 16). Considerable research has focused on the effects of circadian disruption during neurodevelopment. The circadian clock is widely involved in regulating synaptic function, neuronal activity, and behavior. Growing basic and clinical evidence have proved that the disruption of the circadian rhythm may be related to the clinical manifestations of ASD and its pathogenesis (17). Sleep issues may vary among different subtypes and individuals diagnosed with ASD, a significant majority of 50 to 80% of children with ASD experience sleep difficulties, in contrast to the prevalence of less than 30% observed in the general pediatric population (18). Molecularly, mouse mutants with altered clock genes display autistic-like behavior, and dysfunction of circadian molecular marker melatonin contributes to the acceleration of pathogenesis of ASD (19–21). Structurally, the total proteins, size, and number of synapses follow a sleep-wake cycle, and the synaptic mRNA and phosphorylation of synaptic proteins in a time-dependent manner (22–24). All this evidence demonstrates that circadian rhythm are involved in the progression of ASD, supporting that targeting the circadian modulation may be a novel therapeutic approach in the management of ASD. Thus, we primarily review and discuss therapeutic approaches based on the pathological characteristics and circadian signal transduction in ASD at multiple levels, aiming to explore novel strategies for enhancing ASD treatment.

## Components and functions of mammalian circadian clock

**Figure 1:**
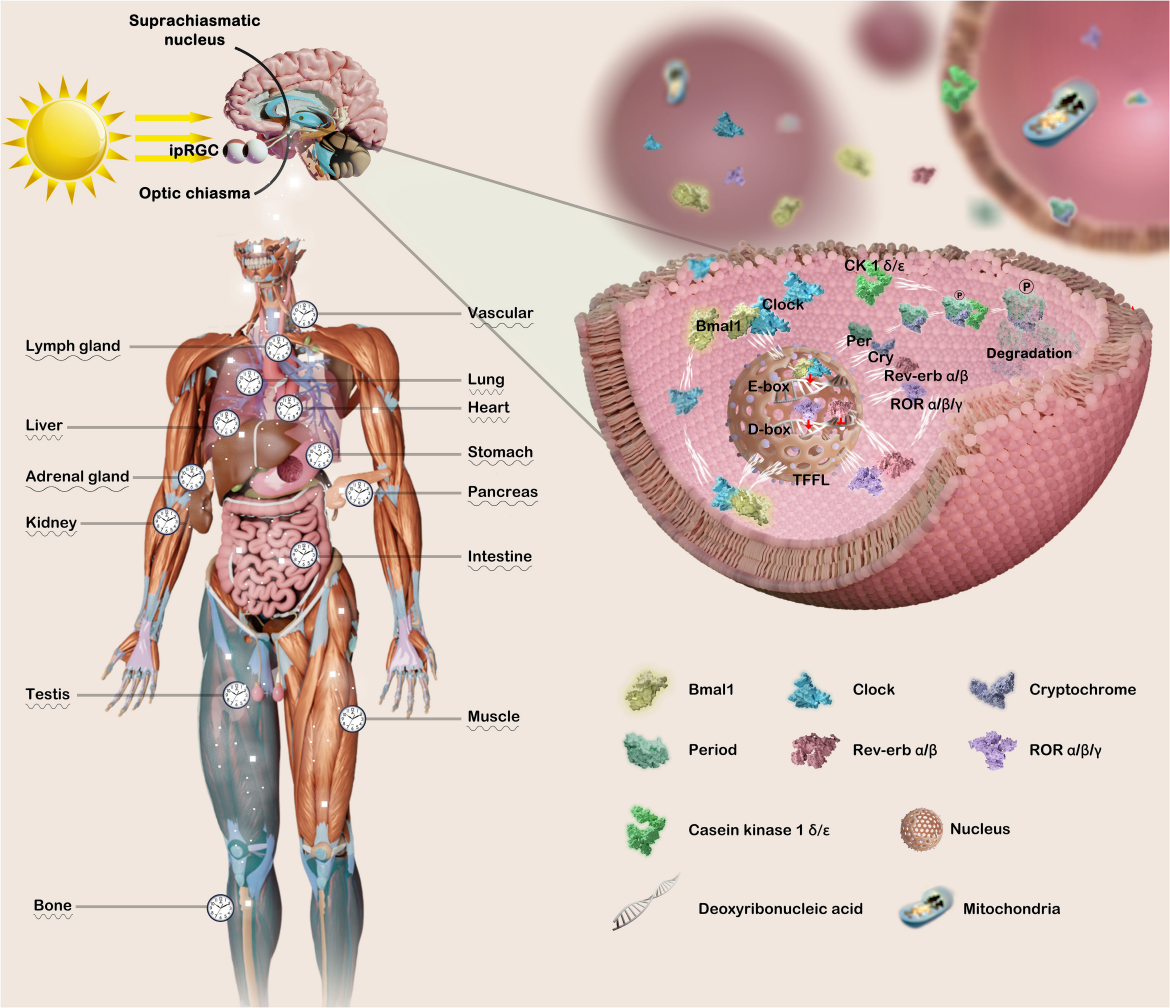
Both the master circadian clock and peripheral clock are regulated by complex and precise molecular mechanisms. The transmission of light triggers the activation of the circadian pacemaker SCN, thereby synchronizing the peripheral clock with the master clock. The coordination of arousal stimulation with the central clock orchestrates the regulation of the body’s physiological processes. The core circadian clock genes Bmal1 and Clock intricately orchestrate the expression of downstream related genes, thereby initiating the transcription process of Per, Cry, Rev-erb, and ROR families. Conversely, Per and Cry infiltrate the nucleus to impede the transcriptional process of Bmal1 and Clock, thereby exerting varying degrees of regulation on numerous physiological and pathological mechanisms governed by the circadian clock. This figure was created by the authors using ‘Blender’ and ‘Photoshop’.

Mammalian circadian physiology is based on hierarchical networks of central and peripheral oscillators. The suprachiasmatic nucleus (SCN) of the hypothalamus, comprising over 20,000 neurons, serves as the primary central pacemaker (25), while also engaging in interactions with peripheral circadian clocks, including those in the heart, lung, liver, stomach, and pancreas (26). Specifically, photosensitive retinal ganglion cells (ipRGCs) containing melanopsin receive external information regarding circadian rhythmic non-visual irradiance, and subsequently transmit this integrated sensory input through various neurotransmitters and hormonal pathways (neurotransmitters involved in light communication, including glutamate, Pituitary Adenylate Cyclase Activating Polypeptide (PACAP), and substance P) and/or neurons (retinohypothalamic tract afferents, RHT) to ventral retinal receptor SCN neurons and peripheral organs and tissues (27) (**Figure 1**).

Molecularly, the Clock and Bmal1 heterodimer translocate to the nucleus where it binds to the enhancer E-box (5′-CACGTG-3′), thereby promoting the expression of *PER1*, *PER2*, *PER3*, *CRY1* and *CRY2*. Conversely, the accumulated Pers and Crys are transported into the nucleus, thereby suppressing the transcriptional activities of *CLOCK* and *BMAL1* mRNA. Subsequent degradation of Per/Cry protein initiates a new cycle of transcriptional activation for *CLOCK* and *BMAL1*, with a duration of approximately 24 hours (28). In the secondary stabilization loop, Clock/Bmal1 heterodimer drive the expression of genes encoding the reverse erythroblastosis virus(*REV-ERBs*, *REV-ERBα/β*) and retinoic acid-related orphan nuclear receptors (*RORs*, *RORα/β/γ*), which respectively repress and activate transcription of *BMAL1* and other clock genes, by binding to the response elements for *RORs* (RORE) on the promoters (29). Furthermore, the PAR-bZip family of circadian transcription factors, including D-box binding protein (DBP), TEF, and HLF, competes with nuclear factor interleukin 3 (NFIL3 or E4BP4) to exert regulatory control over clock-controlled gene expression from D-box-containing promoters (30, 31). In addition, different posttranslational modifications partially govern the circadian process. For example, the stabilization of Per family protein relies on the casein kinase 1 (CK1, e.g., CK1α, CKIϵ), which phosphorylates Per, Cry family protein undergo phosphorylation and degeneration (32–34).

Mutations in these genes result in behavioral and physiological dysfunction, as well as alterations in the period, phase, and circadian rhythm. Interestingly, circadian disruption and poor sleep quality are prevalent in neurodevelopmental disorders, including ASD. Individuals with ASD are relatively common and have been linked to various biomarkers of circadian disruption, such as misaligned cortisol rhythms and lower-amplitude melatonin rhythms (17, 35, 36). What’s more, mutant circadian genes in mice contribute to autistic-like behaviors, some research has reported Bmal1 mutant mice exhibit impaired social interaction and increased anxiety (18, 37). Therefore, in this research, we will investigate the role of circadian physiology in neurodevelopmental processes, provide a comprehensive overview of existing evidence which suggests underlying connections between the circadian and ASD, aiming to explore the novel therapeutic approaches in the management of ASD.

## Links between circadian and ASD

The circadian clock orchestrates a multitude of physiological processes and governs circadian rhythms, such as sleep-wake cycle, by providing timing information for regulation through intricate interactions of endogenous mechanisms and neurohormones (38, 39). When discussing the links between circadian and ASD, several crucial factors must be considered: firstly, the intricate regulatory mechanisms of circadian clock genes; secondly, the effects of inherent rhythmic variations within the circadian system itself; thirdly, the broader implications of circadian regulation on sleep patterns and associated physiological biomarkers should also be incorporated into the analysis. It has been reported that mutations in circadian genes in mice contribute to autistic-like behaviors. Specifically, studies have shown that mutations in the *BMAL1* gene result in several behavioral and neurological anomalies. Affected mice demonstrate impaired social interactions, increased anxiety, immature dendritic spine morphology, enhanced excitatory and inhibitory synaptic transmission, and reduced firing rates (18, 37).

Interestingly, synapses, the specialized junctions between neurons, are fundamental units of brain function, facilitating communication throughout the brain. ASD is typically diagnosed within the first three years of life, a critical period of intense synaptogenesis (40). Synaptic mRNA expression levels demonstrate a circadian pattern, peaking during the initial phase of waking (around dawn) or after mice fall asleep (around dusk). This pattern is reflected in the daily fluctuations of a significant proportion of coding proteins (12%) and over half of phosphoproteins, which show peaks in abundance and phosphorylation status at these key times (23, 24). The diurnal fluctuations in levels of circadian clock proteins, influenced by the time of day, show significant modifications in MT1/2^−/−^ mice compared to their wild-type counterparts. Furthermore, these alterations enhanced spatial learning efficiency during daylight hours (41). Additionally, the direct inhibition of cAMP response element-binding protein (CREB) phosphorylation by G protein-coupled receptors leads to a reduction in the expression of clock proteins Per1 and Per2, thereby impairing the entrainment of the circadian clock by light stimuli (42). *In vivo*, the rhythmic manifestation of Per1 in the pars tuberalis was abolished following pinealectomy, resulting in asynchronous manifestation of Per1 and Per2 in the SCN (43, 44).

Sleep issues may vary among different subtypes and individuals diagnosed with ASD, a significant majority of 50 to 80% of children with ASD experience sleep difficulties, in contrast to the prevalence of less than 30% observed in the general pediatric population (18). The act of sleep plays a pivotal role in the intricate development and maturation of neural pathways, therefore, insufficient sleep can have a significant impact on children’s cognitive abilities, encompassing attention span, memory retention, emotional regulation, and behavioral patterns (45, 46). The development of circadian rhythm sleep-wake disorders (CRSWDs) is influenced by genetic variations in clock and melatonin pathway genes. Significant allelic associations were observed between Polymorphisms of Per1 and Neuronal PAS domain protein 2 (NPAS2) with ASD (47). Notably, in a mouse model, the absence of Npas2^–/–^ resulted in deficits in complex emotional memory and played a crucial role in non-rapid eye movement (REM) sleep homeostasis, leading to decreased total sleep duration in male mice (48, 49). Male and female Shank3^ΔC/ΔC^ mice exhibited significantly reduced sleep duration compared to their wild-type and Shank3^WT/ΔC^ littermates shortly after weaning, with a progressive increase in sleep fragmentation during adolescence (50). Polysomnography (PSG) studies have revealed a decrease in rapid eye movement (REM) sleep, N2 non-rapid eye movement (NREM) sleep, and lower slow wave sleep (SWS) percentage and EEG sleep spindles among individuals with ASD, while an increase in N1 NREM sleep, which indicating evident indications of impaired sleep quality (51). It should note that the detection of sleep parameters similar to those measured by PSG have been confirmed in studies across different age groups of ASD individuals, adolescents and young adults exhibit severe sleep-wake phase disorder, characterized by longer sleep latency, and increased time in bed (TIB) time, while longer sleep onset latency (SOL) and wake after sleep onset (WASO) time (52). Additionally, participants with ASD had significantly lower scores on adolescent sleep-wake scale (ASWS) particularly in areas such as going to bed and falling asleep. Sleep parameters derived from wrist actigraphy also showed longer sleep latency and lower sleep efficiency in ASD individuals (53).

Individuals diagnosed with ASD exhibit diminished levels of melatonin and its primary metabolite, urinary methylmelatonin-6-sulfate, as evidenced in urine, serum, and plasma samples (54). The results of an *in vitro* study demonstrate that melatonin significantly enhances the proliferation of neurospheres and cell viability of induced pluripotent stem cells (iPSCs), while also promoting neural differentiation. The efficient formation of memory and synaptic plasticity heavily relies on the crucial factor of phosphorylating CREB, which is widely recognized as the ‘memory molecule’ (55). Interestingly, in MT_1/2_^−/−^ mice, there was a significant attenuation in the overall amplitude of pCREB compared to WT mice, which contributes to the changes of hippocampal LTP associated with activity-dependent synaptic plasticity (41). Therefore, the correction of melatonin levels through exogenous administration represents a rational therapeutic strategy. Indeed, clinical evidence have demonstrated the profound efficacy of melatonin therapy in ameliorating disrupted sleep patterns in individuals with ASD (56, 57). However, further comprehensive investigations are warranted to elucidate the precise impact of melatonin on synaptic pathophysiology in ASD patients.

## Circadian clocks govern the synapse plasticity in ASD

As previously discussed, sleep disturbances, memory impairments, and temporal dysregulation are all hallmark features of ASD, while the regulation of sleep, memory, and timing is governed by circadian clock genes in various species (58). *De novo* loss-of-function variants have been detected in the clock genes *PER1*, *PER2*, *TIMELESS*, *BMAL1*, and *NPAS2* among individuals with ASD (47, 59, 60). Multiple findings substantiate the role of sleep in facilitating synaptic plasticity, including sleep-mediated enhancement of learning and memory, accompanied by electrophysiological and molecular alterations indicative of synaptic remodeling (61–63). The impact of the sleep-wake cycle and melatonin on synaptic efficacy has been directly assessed through electrophysiological potentials and indirectly evaluated via measurements of plasticity-related mRNAs or proteins, which can modulate synaptic potentiation or depression by altering post-translational modification levels and concentrations (64). Additionally, the circadian biomarker melatonin exhibits neuroprotective properties (65), investigations conducted on the rodent hippocampus have demonstrated that fluctuations in melatonin levels exert a significant impact on synaptic plasticity (66, 67), and long-term potentiation within this brain region (41, 68).

## Synapse formation and function are underlying factors contributing to ASD

**Figure 2:**
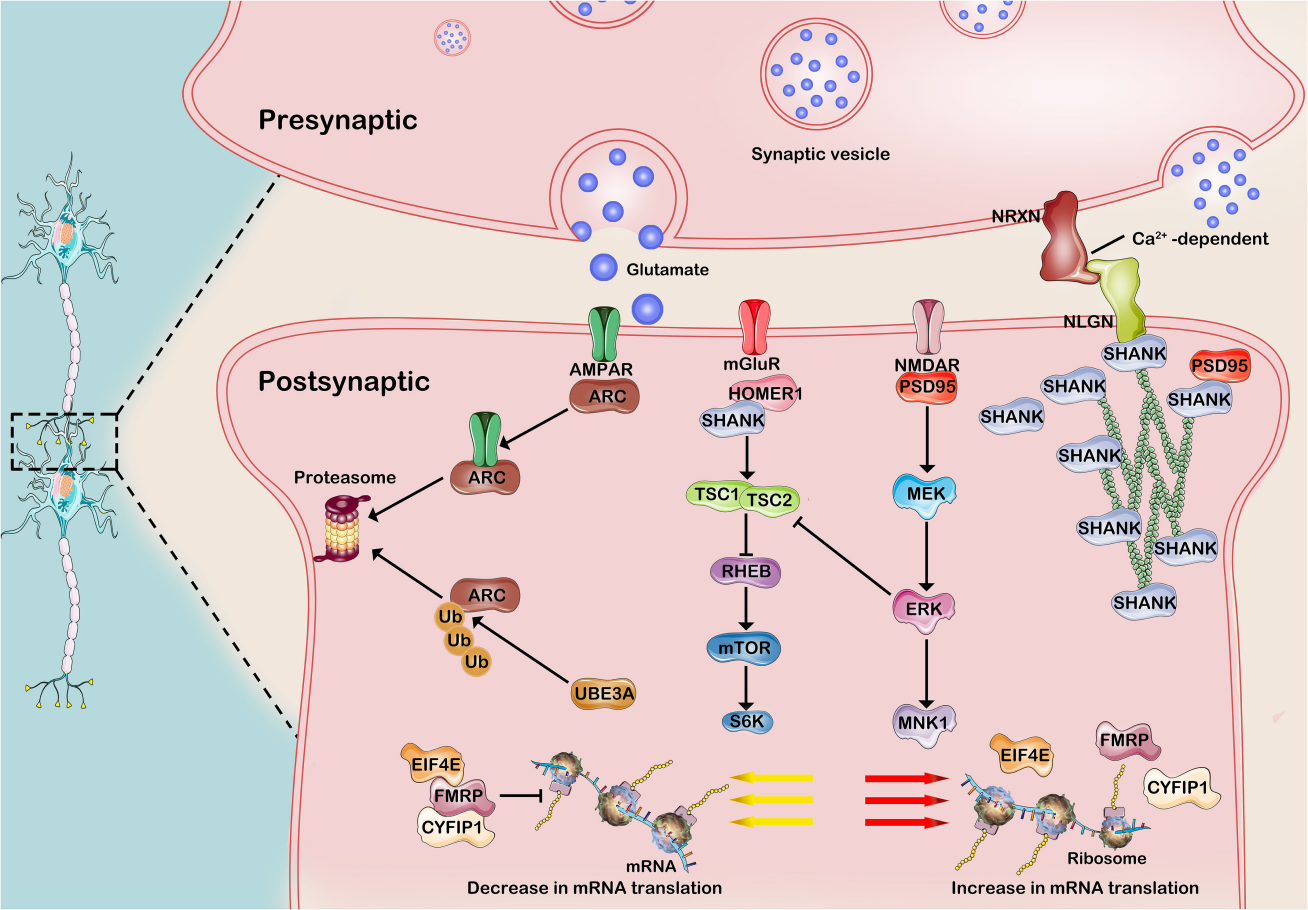
Common molecular mechanisms of developmental synaptopathies associated with ASD. 1)Neuronal activity enhances the transcription of UBE3A and governs the development of excitatory synapses by regulating the degradation of ARC, a receptor that promotes AMPAR internalization in synaptic proteins. The FMRP-EIF4E-CYFIP1 heterotrimer orchestrates the translational dynamics of synaptic mRNA, while its activity is intricately modulated by neuronal stimuli via intricate signaling cascades involving mGluR1, TSC, and mTOR. Cell adhesion molecules, such as NRXNs and NLGNs, along with Shank3 scaffolding proteins, play a pivotal role in bridging glutamate receptors and signaling pathways, exerting influence on scaffolding proteins and the actin cytoskeleton at the postsynaptic density. AMPA-R, a-amino-3-hydroxy -5-methyl-4-isoxazolepropionic acid receptor; mGluR, metabotropic glutamate receptor; NMDA-R, N-methyl-D-aspartate receptor; ARC, activity-regulated cytoskeleton -associated protein; PSD95, postsynaptic density protein 95; SHANK3, SH3 and multiple ankyrin repeat domains 3; Ub, ubiquitin; UbE3A, ubiquitin protein ligase E3A; FMRP, Fragile X Messenger Ribonucleoprotein; EIF4E, eukaryotic translation initiation 4E; CYFIP1, cytoplasmic FMRP–interacting protein 1; RHEB, RAS homologue enriched in brain; TSC, Tuberous sclerosis complex; MEK, MAPK/ERK kinase; ERK, extracellular signal-regulated kinase. This figure was created by the authors using ‘Photoshop’.

Synapses are specialized junctions between neurons that facilitate communication and serve as fundamental units of brain function. It is widely recognized that individuals with ASD are typically diagnosed within the first three years of life, a period marked by intense synaptogenesis (40). Alterations in most rare and *de novo* genes, concentrated within specific biological pathways, have varying impacts on synaptic plasticity and connectivity. These pathways encompass the modulation of chromatin structure, the transcriptional process of genetic information, protein synthesis and degradation, synaptic receptor regulation, cell adhesion molecule function, scaffolding protein support, and actin filament organization within cells (69). Specifically, neuronal activity increases ubiquitin (Ub)–protein ligase E3A (UBE3A) transcription and control the degradation of activity-regulated cytoskeleton-associated protein (ARC) in individuals with ASD. Other transcription factors encoded by ASD-risk genes, such as *TSC, FMRP* and *SHANK3* contribute to early brain development. Additionally, the FMRP–EIF4E–CYFIP1 complex regulates the translation of more than 1,000 genes, many of which are ASD-risk genes. The mTOR and ERK pathways are often hyperactivated in individuals with ASD, leading to increased protein synthesis and abnormal synaptic plasticity, which contribute to the cognitive and behavioral symptoms of the disorder. The alterations in synaptic efficacy and subsequent changes in neuronal connections are influenced by external stimuli, sensory input, and internal brain activity through the modulation of synapse numbers (69, 70). Before delving into the involvement of the circadian system in synaptopathology pathogenesis, it is imperative to clarify ASD-associated risk genes implicated in synaptopathies (**Figure 2**).

### Electrophysiological modulation of synaptic function by circadian

The changes in the frequency of excitatory postsynaptic potential slopes (EPSPs) reflect modifications in the presynaptic component of synaptic transmission, while alterations in amplitude indicate adjustments in the postsynaptic component (22). In 1984, Hanada and Kawamura first reported the presence of a circadian rhythm in both averaged postsynaptic and presynaptic evoked responses *in vivo*, which exhibited independence from the state of vigilance and could be abolished by bilateral SCN lesions (71, 72). In subsequent *in vitro* studies, the persistence of the time-of-day effect for extended durations in the ex vivo state strongly implies that the hippocampus itself exhibits circadian properties in synaptic efficacy (73–75). The parameters in the cerebral cortex of mice and rats exhibit decreased values following a few hours of sleep, increased values after a few hours of wakefulness, and demonstrate a decline during recovery sleep subsequent to sleep deprivation (76). The phenomenon of orientation-specific response potentiation (OSRP) is considered an *in vivo* manifestation of long-term potentiation (LTP) at glutamatergic synapses, resulting in an enhanced response to a specific visual stimulus (77). The occurrence of OSRP is specifically observed during the entrained circadian sleep phase and further consolidated during post-stimulus sleep (78, 79). Given that the impact of time of day on learning memory, and hippocampal LTP in Light: Dark entrained conditions (74, 80), subsequent investigations have reported decreased EPSPs in Bmal1 KO mice, indicating impaired long-term synaptic plasticity (81).

In MT_1/2_^−/−^ mice and WT-SCGX(wild-type mice that underwent bilateral surgical removal of their superior cervical ganglia) mice, the circadian pattern of LTP is abolished, which suggests that melatonin significantly influences the circadian synaptic plasticity in mouse hippocampus in the activity-dependent manner (41). The administration of melatonin prior to the recording of patch-clamp data exhibited a significant restoration of both the frequency and amplitude of spontaneous EPSCs observed in hippocampal neurons following sevoflurane (Sev) treatment (82). Additionally, melatonin pretreatment significantly enhanced dendritic branching in pyramidal neurons and upregulated the expression of synaptic scaffold proteins (Homer, PSD-95) compared to Sev-treated mice (82). Chronic melatonin treatment effectively restored LTP in Ts65Dn (TS) mice, a Down syndrome (DS) model (83). These results demonstrate the significant impact of melatonin on normalizing synaptic LTP and mitigating morphological neurodivergence, as evidenced by both electrophysiological and neuromorphological analyses. These findings suggest that melatonin holds promise as an effective therapeutic intervention for delaying the progression of neuropathology associated with ASD individuals.

### Molecular mechanism linking circadian and synaptic function

The transport of GluA1-containing AMPARs in and out of the synaptic membrane is the principal mechanism underlying synaptic potentiation and depression, respectively. The AMPARs exhibit high calcium permeability, and their expression shows a superlinear relationship with the size of the postsynaptic density, thereby conferring significant influence on synaptic strength (84). The levels of GluA1-containing AMPARs are 30-40% higher following wakefulness compared to sleep in rats, indicating a state of net synaptic potentiation during wakefulness and depression during sleep. Additionally, the phosphorylation patterns of AMPARs, as well as the enzymes CaMKII and GSK3β, align with these findings (85). This implies that the variability in synaptic efficacy during sleep and wakefulness is attributed to alterations at the postsynaptic level, as previously indicated by changes in OSRP (85). Several reports indicated that synaptic Shank3 protein exhibits minor oscillations during the day in the hippocampus and striatal brain region that correlate with the altered level of serum melatonin (86). Further, the concentration of Shank3 increases rapidly during wakefulness by inducing the active phase (86). The research findings have confirmed that the lack of FMRP protein results in excessive growth of dendritic branches, enlarged synaptic terminals, and impairments in developmental and activity-based trimming (87–89).

Indeed, the levels of synaptic plasticity-related proteins are associated with a reduction in synaptic efficacy following sleep. For instance, the levels of phosphorylated CREB protein, Arc, and BDNF - three genes commonly associated with the induction of synaptic plasticity - exhibit an increase during wakefulness but decline following prolonged periods of sleep (90). Dark rearing increased BDNF protein levels in the primary visual cortex region, but decreased *BDNF* mRNA levels (91). It is evident that circadian rhythms do not always synchronize the transcription and translation of synaptic plasticity-related mRNAs, and the impact of sleep-wake cycles on these processes lacks consistency. The synaptic scaffold proteins (e.g. gephyrin and PSD-95) and neurexin 2α in the SCN region of C3H/J mice exhibit a diurnal rhythm, peaking at ZT14 and subsequently declining during the dark period to reach minimal levels during the day (92). In subsequent experiments conducted in SCN2.2 (immortalized rat SCN cells), melatonin treatment was found to enhance the amplitude and phase changes of synaptic proteins PSD-95 and gephyrin, demonstrating its capacity to induce phase shifts on these proteins similar to its effect on core clock components such as Per2 (92, 93).

### Structural changes in synapses mediated by circadian

Gene mutations contribute to synaptic plasticity by modulating the strength and/or quantity of synapses. Furthermore, gene mutations have also been observed to exert an impact on neuronal networks in animal models, leading to either excessive activity and increased synaptic density or insufficient activity and decreased synaptic density. Surprisingly, many circadian mutations, particularly those in Bmal1, lead to enhanced gene transcription, mRNA translation and protein synthesis, effects that are also observed in response to alterations in neuronal activity (69). Mutations in negative regulators of mTORC1, including *TSC1*, *TSC2*, and *PTEN*, are associated with the development of TSC, FXS, AS, RTT (94, 95). *In vivo*, mutations in *TSC1/2* result in circadian rhythm irregularities due to dysregulated proteostasis of the core circadian clock protein Bmal1 (96). *In vitro*, Bmal1 determine the expression of synaptic plasticity−related protein, including BDNF, synpsin1, and synaptotagmin1 (97). This observation is in line with the finding that the circadian oscillation of protein synthesis rate is under the control of Bmal1, a process regulated by ribosomal S6 protein kinase 1 (S6K1), a critical translational regulator of mTOR effector protein kinases, which rhythmically phosphorylates Bmal1 at evolutionarily conserved sites (98).

In Bmal1 KO and Bmal1^flx/flx^: L7-Cre mice, several behavioral deficits were observed, including impaired sociability as well as increased behavioral stereotypy in the open field and three chamber tests, when compared to wild-type mice (18, 37). The cerebellar Purkinje cells (PCs) in Bmal1 KO and Bmal1^flx/flx^: L7-Cre mice exhibited heightened activation of mTORC1 and displayed a nascent dendritic spine morphology. Specifically, there was an increased proportion of immature dendritic spines and a decreased proportion of mature dendritic spines in the PCs (37). In addition, the shape of dendritic spines exhibit a significant diurnal and circadian changes, which that are differentially regulated: single-synapse spines remain under circadian clock regulation, while changes of double-synapse spines are driven by light (99). These electrophysiological alterations observed in Bmal1^-/-^ mice, including enhanced excitatory and inhibitory signaling and amplified levels of oscillatory activity, align harmoniously with the observed underdeveloped structure of dendritic spines resulting from mechanisms occurring subsequent to synaptic transmission (37) (see **Table 1**).

**Table 1:** Evidence contributes to circadian clocks govern the synapse plasticity in ASD.

Sort	Target	Outcome	Reference
Electrophysiological	fEPSP	The slope and duration of fEPSP were increased in mouse hippocampal slices in subjective night compared to the day.	(74)
	fEPSP	The slope of fEPSP in the CA1 synapse in hippocampal slices exhibited an increase level at CT0 compared to CT12.	(100)
	fEPSP	MT_1/2_^−/−^ mice and WT-SCGX mice, the circadian pattern of fEPSP was abolished, suggesting that circadian significantly influence the circadian synaptic plasticity in mouse hippocampus.	(41)
	fEPSP	Circadian biomarker melatonin increase the amplitudes of fEPSP in hippocampal neurons.	(83)
	mEPSCs	The frequency and amplitude of mEPSCs from frontal cortex slices of mice and rats increased after waking and decreased after sleep, independent of time of day.	(76)
	EPSCs	Circadian biomarker melatonin restores both the frequency and amplitude of spontaneous EPSCs in hippocampal neurons.	(82)
	OSRP	OSRP was only evident after subsequent sleep and promote cortical synaptic potentiation *in vivo*, while blocked by sleep deprivation.	(78)
Molecular	Bmal1	pBmal1-S42A contribute the synapse plasticity and is required for the circadian rhythm of hippocampal plasticity.	(100)
	PSD95	Melatonin pretreatment enhanced dendritic branching in pyramidal neurons and upregulated the expression of PSD-95.	(82)
	PSD95	Circadian biomarker melatonin enhances the amplitude and phase changes of PSD-95 and gephyrin.	(92, 93)
	Bmal1, PSD95	BMAL1 deficiency decreases microglial synaptic PSD95 engulfment in aged mice.	(101)
	Shank3	The concentration of Shank3 increases rapidly during the wakefulness by inducing the active phase.	(86)
	pCREB, Arc, and BDNF	Synaptic plasticity associated molecular exhibit an increase during wakefulness but decline following prolonged periods of sleep.	(90)
Structural	Dendritic spines morphology	Bmal1^flx/flx^: L7-Cre mice exhibited a nascent dendritic spine morphology, specifically, increased proportion of immature dendritic spines and a decreased proportion of mature dendritic spines.	(18, 37)
	Dendritic spines morphology	Shape of dendritic spines exhibit a significant diurnal and circadian changes: single-synapse spines remain under circadian clock regulation, while changes of double-synapse spines are driven by light.	(99)
	Spine number	The number of spines follow the circadian fluctuation pattern which contribute to learning capacity in the hippocampus.	(102, 103)

## Towards the development of treatment

### Reaching the management of treatments for autism

**Figure 3:**
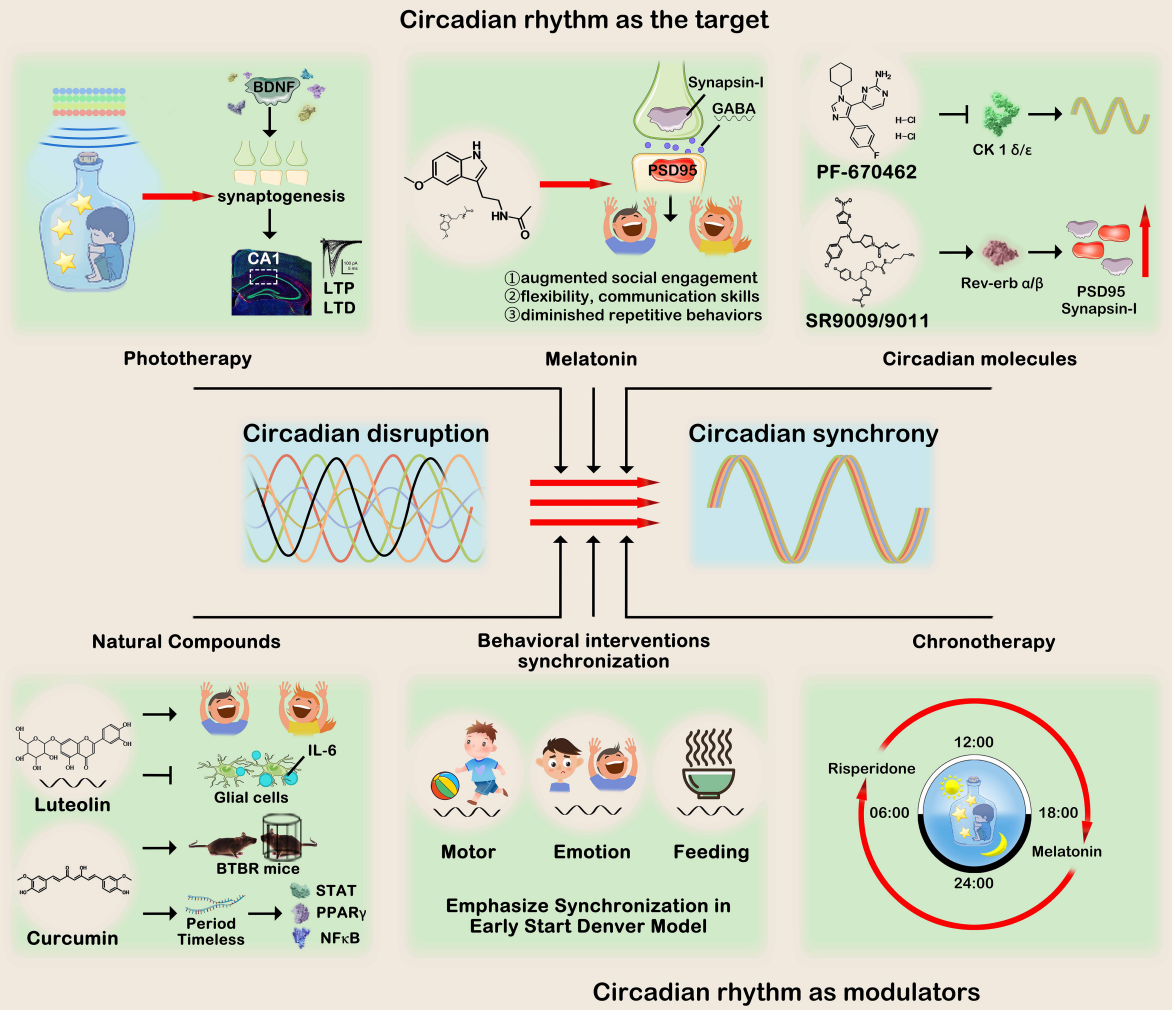
Circadian rhythm treatments for ASD can be roughly divided into two categories:1) Circadian rhythm as the target and 2) Circadian rhythm as the modulator. The first category encompasses phototherapy, melatonin supplementation, circadian rhythm modulators, and natural compounds. Their collective objective is to regulate pivotal molecules involved in circadian rhythms, thereby improving autistic-like behaviors symptoms. The second category utilizes circadian rhythms to optimize clinical behavioral intervention or medication administration, emphasizing the synchronization of motor and emotions, as well as the precise timing of drug consumption. This figure was created by the authors using ‘Photoshop’.

As previously mentioned, the circadian system, comprising central and peripheral clocks, can be influenced by a range of environmental and systemic physiological factors, including light exposure, feeding patterns, hormone regulation, and physical activity. Utilizing this rhythm synchronizer can enhance the efficiency of circadian clock operation, improve circadian rhythm and potentially decreasing vulnerability to diseases affected by internal desynchrony. The profound impact of circadian rhythms on physiology gives rise to two primary approaches for translating prior knowledge of this mechanism into clinical application. Chronotherapy can be classified into two categories(see **Table 2**): (1) targeting biological rhythms by using drugs or non-pharmacological interventions to manipulate circadian rhythms or core clock genes; and (2) utilizing endogenous rhythm regulation drugs established by the biological clock as regulators of therapeutic drugs, which can improve efficacy and reduce adverse reactions (125). In the subsequent sections, we delineate therapeutic applications of both non-pharmacological and pharmacological interventions that target circadian clocks in disease contexts (**Figure 3**).

**Table 2:** Towards the management of treatments for autism.

Sort	Method	Results	Reference
Phototherapy	Transcranial light therapy (TLTC)	TLTC upgrades LTP and LTD in the CA1 collateral pathway.	(104)
	low-level laser light therapy (LLLT)	LLLT ameliorate irritability, stereotypic behavior and other symptoms.	(105)
Melatonin	Oral administration of 1mg melatonin for 3 weeks, if there is no response, then gradually increase to 9mg in the following 13weeks	Melatonin yields favorable outcomes in terms of autism-like behaviors (such as augmenting social engagement, flexibility, communication skills, diminishing repetitive actions or anxiety.	(57, 106)
	Oral administration of 3mg melatonin before bedtime	Melatonin significantly reduces screaming attacks and improves sleep quality, with onset of sleep occurring within 30 minutes in RTT individuals.	(107)
	10^−5^ mol/L melatonin *in vitro* and i.p.10mg/kg melatonin *in vivo*	Melatonin treatment increase presynaptic activity marker Synapsin-I and Postsynaptic protein marker PSD95.	(82, 108)
	Melatonin *in vivo*	Melatonin enhances GABAergic transmission in the brain and modulate diurnal variations.	(109, 110)
Circadian components	PF-670462	PF-670462 administration rescue memory deficit and normalized behavior.	(111–113)
	SR9009, SR9011	SR9009/SR9011 administration induces wakefulness, inhibits sleep, regulates emotional behavior, and mitigate anxiety-related behaviors.	(114)
	SR9009	SR9009 administration enhances the expression of pre- and post-synaptic markers PSD-95 and synaptophysin.	(115)
Natural Compounds	Luteolin formulation	Luteolin formulation enhances eye contact and attention, increase social interaction, and resume speech.	(116)
	Luteolin	Luteolin safeguards synaptic function and enhances memory, as evidenced by its facilitation of synaptic transmission and induction of long-term potentiation.	(117)
	Neonatal administration of curcumin	Curcumin enhances sociability, reduce repetitive behaviors, and improve cognitive impairments.	(118, 119)
Circadian modulators	Melatonin administered in the late afternoon	Melatonin advances the circadian rhythm of physiology and behavior when administered in the late afternoon.	(120)
	Risperidone and melatonin administered in different phase	Administered risperidone in the morning and melatonin at 21:00 during their inactive phase exhibited enhanced social interaction and improved occupational status	(121)
	Aripiprazole administered in the morning and evening	Aripiprazole administered in the morning and evening modulate its metabolic side effects, either reducing them.	(122)
	Synchronize the ESDM	therapist will replicate the rhythmic motor pattern of ASD child precisely with a corresponding rhythmic pattern.	(123, 124)

### Circadian clocks as the target

#### Phototherapy

While there is currently no FDA-approved treatment or light device specifically designated for this purpose, extensive research has been conducted on the utilization of light to synchronize circadian rhythms. Light exposure has been widely acknowledged as a therapeutic intervention for sleep disorders, such as advanced or delayed sleep phase syndrome, and ASD, which are characterized by genetic, environmental, and pathological disruptions of circadian rhythms (126). Transcranial photobiomodulation (PBM), which is characterized by low level light therapy, has been demonstrated to confer cognitive benefits by modulating mitochondrial function, reducing inflammation, stimulating neurogenesis and synaptogenesis in the cortex, hippocampus, and subventricular zone, thereby facilitating memory enhancement and improved learning abilities (127). In a 5-days consecutive experiment, transcranial light therapy (TLTC) device delivers a constant light intensity of 74.5 lux during the 125 seconds the light module is on, results demonstrated that light therapy upgrades LTP and LTD in the CA1 collateral pathway (104). Moreover, low-level laser light therapy (LLLT) has been demonstrated as an efficacious intervention for reducing irritability, lethargy/social withdrawal, stereotypic behavior, hyperactivity/noncompliance, inappropriate speech and other symptoms and behaviors associated with ASD in children and adolescents, exhibiting sustained positive outcomes over an extended period of time (105). The promoting effect observed may be attributed to the stimulation of BDNF production, which in turn facilitates synaptogenesis (128). Cumulatively, these investigations suggest that light-based strategies potentially confer advantages in the prevention and management of ASD.

#### Melatonin

Since 1993, researchers have been studying the effects of melatonin supplementation in individuals diagnosed with ASD (129). Furthermore, recent treatment consensus guidelines recommend the inclusion of melatonin as part of the treatment plan (130, 131). Multiple studies have demonstrated that melatonin decreased sleep latency and improved sleep duration (132, 133). Although the melatonin cycle is not considered a pivotal component of the core circadian clock, strategically targeting implies that leveraging the circadian output pathway for medication could potentially offer a more advantageous approach compared to focusing solely on essential components of the core circadian clock (125, 134). In clinical studies, three recent major studies conducted in the United States, France and England aim to demonstrate the efficacy of melatonin by examining the impact of three different doses on alleviating sleep disorders (NCT01993251, NCT00927030, NCT01780883). Furthermore, the administration of melatonin supplements may potentially yield favorable outcomes in terms of autism-like behaviors (such as augmenting social engagement, flexibility, communication skills, diminishing repetitive actions or anxiety) (57, 106). Oral administration of three milligrams of melatonin before bedtime resulted in a significant reduction of screaming attacks and improvement in sleep quality, with onset of sleep occurring within 30 minutes in RTT individuals (107).

Several studies have elucidated the pivotal role of melatonin in facilitating synapse formation through a receptor-mediated mechanism. Furthermore, these investigations have provided compelling evidence suggesting that melatonin can augment both the quantity and rate of secretion in axonal and somatic cells. This was corroborated by conducting immunostaining experiments employing the Synapsin-I as a marker for presynaptic activity and the Postsynaptic Density-95 (PSD95) protein marker (82, 108). Given the implication of an imbalance in the excitatory (Glutamatergic) and inhibitory (GABAergic) neurotransmitter systems in the pathogenesis of ASD (135), it is crucial to consider the precise equilibrium between neurotransmitter and receptor production during this developmental period spanning from 12 to 30 months (136). *In vivo*, melatonin has been demonstrated to enhance GABAergic transmission in the brain and modulate diurnal variations (109, 110). Given the established efficacy and absence of long-term toxicity, melatonin therapy emerges as a highly safe chronobiotic strategy for managing sleep architecture and synapse formation in autistic individuals.

#### Modulating circadian components

In recent years, the discovery of small molecule modulators of circadian rhythms targeting core or non-core circadian components has expanded circadian treatment options for individuals with ASD. Although no reports have been published on small molecule therapeutic targets specifically targeting circadian rhythm in ASD, it is important to consider that like most pharmacological agents, small molecule agents may have additional non-circadian targets and can induce significant changes in key ASD-related phenotypes, which would be crucial for enhancing efficacy. Given its potential to modulate the circadian clock broadly, PF-670462, a small molecule that can penetrate the blood-brain barrier and inhibit CK1α/ϵ (137), shows promise in stabilizing circadian rhythms in various mouse models of circadian dysfunction. This compound may also have the ability to mitigate proteomic alterations, cognitive performance deficits, and disturbances in the sleep-wake cycle associated with neurodegenerative diseases (111–113). Pharmacological targeting of REV-ERBs exhibits promising potential for therapeutic intervention in a broad range of neurological, metabolic, and immune disorders. Compounds believed to function as REV-ERB agonists, such as SR9009 and SR9011, have demonstrated their capacity to induce wakefulness, inhibit sleep, regulate emotional behavior, and mitigate anxiety-related behaviors in murine models (114). Moreover, SR9009 was confirmed to enhance the expression of pre- and post-synaptic markers PSD-95 and synaptophysin through modulating synaptic pruning, thereby ameliorating synaptic deficits and improving cognition impairment in neurodevelopmental diseases (115).

#### Natural compounds

In addition to synthetic compounds, some natural chemical substances also have the function of regulating the biological clock, providing a theoretical possibility for the concept of chrono-medicine in the prevention and treatment of ASD. The natural plant flavonoid, luteolin, effectively attenuated IL-6 expression in glial cells and demonstrated neuroprotective and anti-inflammatory properties (138). Administration of a luteolin formulation (NeuroProtek^®^) in conjunction with conventional medication resulted in significant improvements among children with ASD, including a 50% enhancement in eye contact and attention, a 25% increase in social interaction, and approximately 10% experiencing the resumption of speech (116). Therefore, luteolin was employed for the management of autistic behavior and enhancement of social behavior (139, 140). The pharmacological effect of luteolin exhibits a diurnal pattern, recent study demonstrates that luteolin induces antioxidant enzymes by activating Nrf2 in the liver of mice at ZT12 (active phase), but not at ZT0 (inactive phase) (141). Furthermore, luteolin demonstrates significant potential in safeguarding synaptic function and enhancing memory, as evidenced by its facilitation of synaptic transmission and induction of long-term potentiation (117).

Neonatal administration of curcumin was found to ameliorate autism-related symptoms in BTBRT^+^ltpr3^tf^/J (BTBR) mice, a well-established model for autism, by enhancing sociability, reducing repetitive behaviors, and improving cognitive impairments (118, 119). Notably, curcumin modulates multiple signaling pathways involved in regulating intracellular timing cycles that generate circadian rhythms. These targets encompass *STAT*, *PPARγ*, and *NFκB*, which exert their effects on gene expression (including mRNA expression of *Period* and *Timeless*) within the interconnected molecular timing loops of the circadian oscillator responsible for rhythm generation (142, 143). Therefore, this also provides us with a potential avenue for future research, exploring whether the synaptic morphology, pathological changes, and behavioral improvements induced by natural compounds in patients with ASD are influenced by circadian rhythms. Specifically, investigating whether the efficacy of natural drugs is time-dependent within a specific period (e.g., active versus inactive periods) would be valuable.

## Circadian rhythm as modulators

Despite the prevailing practice of administering medicines without considering the timing, comprehensive circadian transcriptome studies have revealed that most drug targets exhibit rhythmic expression in specific tissues (144, 145). For example, melatonin advances the circadian rhythm of physiology and behavior when administered in the late afternoon, while it delays it in the early morning (120). In randomized controlled trials, both risperidone and aripiprazole, FDA-approved medications have demonstrated efficacy in alleviating symptoms of irritability or agitation in children and adolescents with ASD (146, 147). Intriguingly, patients with neurodevelopmental disorders who were administered risperidone in the morning and melatonin at 21:00 during their inactive phase exhibited enhanced social interaction and improved occupational status (121). Additionally, aripiprazole exhibits a significant influence on circadian behavior and cellular rhythm within the SCN. *In vivo*, chronic administration of aripiprazole enhances entrainment to the external light-dark cycle, while in an experiment utilizing SCN slice cultures, the application of aripiprazole disrupts cellular synchrony, thereby attenuating the rhythm (148). Indeed, a 14-year longitudinal study demonstrated that the dosing schedule of aripiprazole (administered in the morning and evening, respectively) may modulate its metabolic side effects, either reducing them (122). Given that metabolic side effects pose significant limitations to antipsychotic usage in individuals with ASD, mitigating this risk would represent a noteworthy advancement.

Developmental behavioral interventions, such as Early Start Denver Model (ESDM) also emphasize the circadian rhythms and synchronization (149, 150). Within the model, the therapist collaborates with the child to establish and maintain synchronized, coordinated activity routines that incorporate teaching opportunities. The ESDM acknowledges the significance of motor, emotional, feeding, and relational synchrony in facilitating early development among children diagnosed with autism. As a comprehensive, behavioral, and developmental early intervention program designed for infants and toddlers diagnosed with ASD, ESDM is grounded in an understanding of circadian rhythmicity and synchrony at multiple levels. For instance, if the child employs an object exhibiting a rhythmic motor pattern, the therapist will replicate this behavior precisely with a corresponding rhythmic pattern. Subsequently, the child’s progress is facilitated by introducing novel and diverse rhythms (123). Furthermore, the therapist’s adjustment of their behaviors, movements, level of arousal, and posture to align with those of the child contributes to establishing fundamental consistencies that are crucial during the initial phase (124).

## Future perspectives and conclusions

In summary, a comprehensive review of previous studies indicates that the influence of biological rhythms on neurodevelopment and synaptic strength in ASD is not characterized by a simple, unidirectional effect. Instead, it appears to be contingent upon multiple factors. These factors encompass the examined circuit, animal age, pre-sleep stimulation type, hormone levels, sleep-wake cycles, and presence of a robust circadian rhythm (oscillation amplitude). Circadian rhythms, whether originating from the central pacemaker or induced by peripheral oscillators, exert a profound influence on synaptic potency and morphology. The determination of the respective roles of state-dependent and clock-dependent alterations in synaptic efficacy represents a crucial area for future research. The state-clock model provides a theoretical framework to investigate these phenomena, as previously acknowledged.

The circadian clock plays a crucial role in regulating key physiological processes during mammalian neural development and has been extensively investigated. Emerging evidence suggests that these two systems can interactively modulate each other through diverse mechanisms in both normal and pathological conditions. Neurodevelopment in individuals with ASD is a highly intricate process, wherein synaptogenesis and the establishment of mature synaptic connections play a pivotal role. Therefore, gaining a comprehensive understanding of the intricate interplay between the circadian clock and synaptic and neurodevelopmental cycles will greatly contribute to the development of efficacious chronotherapeutic interventions for ASD.
